# Right Place, Right Time: Preferences of Women with Ovarian Cancer for Delivery of CAM Education

**DOI:** 10.3390/medicines2030236

**Published:** 2015-08-31

**Authors:** Judith Ann Ebbert, Kristine A. Donovan, Cecile A. Lengacher, Donna Fabri, Richard Reich, Ellen Daley, Erika Lynne Thompson, Robert M. Wenham

**Affiliations:** 1 H. Lee Moffitt Cancer Center & Research Institute, 12902 Magnolia Drive, Tampa, FL 33612, USA; E-Mails: kristine.donovan@moffitt.org (K.A.D.); donna.fabri@moffitt.org (D.F.); robert.wenham@moffitt.org (R.M.W.); 2 University of Florida Health, College of Nursing, 12901 Bruce B. Downs Blvd., Tampa, FL 33612 USA; E-Mail: clengach@health.usf.edu; 3 Division of Psychology, College of Arts and Sciences, University of South Florida Sarasota-Manatee, Tamiami Trail Sarasota, FL 34243, USA; E-Mail: rreich@sar.usf.edu; 4 University of Florida College of Public Health, 13201 Bruce B. Downs Blvd., Tampa, FL 33612 USA; E-Mails: edaley@health.usf.edu (E.D.); ethomps1@health.usf.edu (E.L.T.)

**Keywords:** ovarian cancer, complementary and alternative medicine, CAM education, feasibility pilot

## Abstract

The purpose of this pilot study was to assess the feasibility of on-site complementary and alternative medicine (CAM) education sessions to maximize quality of life for women with ovarian cancer. The pilot intervention consisted of four weekly sessions, each focusing the techniques and benefits of a particular CAM topic (e.g., nutrition, massage, relaxation). Participants were recruited from the Center for Women’s Oncology at H. Lee Moffitt Cancer Center from 2010 to 2012. Eligible participants had an ovarian cancer diagnosis with a life expectancy of at least 12 months, and were 18 years or older. The Gynecologic Oncology research nurse invited women in the outpatient clinic who matched the eligibility criteria. The research nurse explained the study and provided an informed consent form and return envelope. Because ovarian cancer is not only a rare cancer but, also, most patients seen at Moffitt have recurrent or advanced disease, many women did not have an adequate ECOG score. Many women who consented had rapid changes in health status, with morbidity and mortality outpacing recruitment of the 20 needed to proceed with the four education sessions. Baseline and follow-up surveys were conducted to assess changes in QOL, knowledge, and satisfaction with the intervention. While 27 women consented and 24 women completed the baseline survey, only five women participated in the intervention. The five women who participated were all white, and at time of consenting had a mean age of 60 (SD 9.08) and an average of 102 months (SD 120.65) since diagnosis, and were all on active treatment, except for one. The intervention pilot did not encounter difficulties with regard to recruitment, but suffered problems in achieving an adequate number of women to launch the on-site sessions because of rapidly changing morbidity and significant mortality. The team recognized that a larger-scaled intervention comprised of on-site sessions was impractical and compared attendance rates with a more convenient format currently underway in the Women’s Oncology program at Moffitt. While low participation prevented an intervention analysis of scientific merit, the study data is informative with regard to barriers, facilitators, and alternative methods for sharing useful information to women with advanced ovarian cancer. The comparison strongly suggested that CAM education for women compromised by the disease and treatment associated with ovarian cancer would best be delivered in the convenient-access format that allowed remote access to live and recorded discussions of specific topics.

## 1. Introduction

Estimated new gynecologic cancer cases in 2013 totaled 91,730; although ovarian cancer comprised 24% of those diagnoses, it accounted for 50% of gynecologic cancer deaths that same year [1]. Ovarian cancer causes more deaths than any other cancer of the female reproductive system, and will cause about 14,270 deaths in the United States during 2014 [2]. Because there are neither proven primary prevention methods nor definitive methods of early detection of ovarian cancer [3], only 19% of women are diagnosed at stage I, which has the highest rate of survival at 94% [4]. Most women with ovarian cancer undergo primary cytoreductive (debulking) surgery followed by postoperative chemotherapy consisting of 6–8 cycles of a platinum/taxane-based chemotherapy regimen [5]. Despite an overall complete response rate to chemotherapy of 75%, most women will eventually experience relapse of their disease [6]. Although many women will receive many additional lines of chemotherapy, relapsed disease is ultimately fatal. Therefore, the impact of treatment on quality of life (QOL) is fundamentally important to these patients.

Women with ovarian cancer are challenged by disease- and treatment-induced side effects that comprise physical, psychological, sexual, social, and spiritual dimensions [3]. More than 30% of women experience alopecia, weight changes, sexual dysfunction, constipation, fatigue, peripheral neuropathy, neutropenia, anemia, thrombocytopenia, diarrhea, mucositis/stomatitis, nausea, hand-foot syndrome, anorexia, edema, or hot flashes [7]. Psychological distress is caused by disruptions in patients’ lives [8], fear of recurrence [9,10], loss of ability to work, financial concerns [11], depression, and anxiety [10,12,13,14,15,16]. Social impairment may include difficulties with vocational, domestic, and sexual functioning [13]. Changed relationships with family and friends and tenuous connections with healthcare providers may produce feelings of abandonment or loneliness [14,17,18,19].

Despite the severe side effects of treatment associated with ovarian cancer, Donovan and colleagues found that women preferred salvage chemotherapy to palliative care, opting for the hope of quantity over QOL [20]. Because QOL for women with ovarian cancer correlates with the number of physical symptoms experienced [15,21,22], and because most women are likely to pursue salvage therapy [20], it is important to test interventions that may allow women to pursue quantity of life without having to sacrifice QOL.

One potential mechanism that can improve QOL is the use of complementary and alternative medicine (CAM). CAM includes a wide array of passive and active mind-body interventions, nontraditional pharmacologic and biologic therapies, and dietary and nutritional strategies [23], only a few of which have been tested in women with ovarian cancer. In a study of CAM use to relieve symptoms, side effects, and psychological distress in women with breast cancer, Lengacher and colleagues found that patients who had undergone chemotherapy were most likely to use CAM [24]. The highest CAM use among study participants was diet and nutrition supplements (vitamins and minerals at 63% and antioxidants at 33%). Two-thirds of the participants reported using at least one stress-reducing technique on a regular basis with prayer/spiritual healing being the most common (49%), followed by support groups (37%), and humor therapy (21%). Several women in the study used massage therapy at least once. Stress-reducing techniques and traditional medicines were reported as more helpful in fighting breast cancer compared to diet and nutritional supplements [24,25].

Very few studies exist for interventions that integrate CAM to improve QOL in women with ovarian cancer. Relaxation training during chemotherapy for ovarian cancer positively affected the immune system [26]. Selenium had beneficial effects on diarrhea in women undergoing pelvic radiotherapy for gynecologic cancer [27,28] and on oxidative stress and the glutathione peroxidase system in women undergoing chemotherapy for ovarian cancer [29], and the use of antioxidants such as vitamins C and E, beta-carotene, and coenzyme Q-10 appeared to improve the efficacy of first-line chemotherapy in two cases of ovarian cancer [30]. Dietary supplements such as vitamin E, alpha-lipoic acid, and glutamine have shown promise in improving peripheral neuropathy, which is a common side effect of chemotherapies used to treat ovarian cancer [31,32,33]. To continue to accelerate advances, the Institute of Medicine has stated that CAM should undergo rigorous testing [34].

The purpose of this pilot study was to assess the feasibility of a pilot study to provide on-site education sessions that focused on CAM services with the potential to maximize QOL for women with ovarian cancer. The pilot feasibility study aimed to: (1) estimate the prevalence of use of various CAM therapies, and identify specific CAM therapies of greatest interest to women with ovarian cancer; (2) identify factors associated with use of CAM therapies among women with ovarian cancer; and (3) assess whether participation in a CAM education programs had an impact on knowledge, intention, and satisfaction.

## 2. Material and Methods

### 2.1. Sample

The target population was adult women with ovarian cancer receiving gynecologic oncology care in the Center for Women’s Oncology at H. Lee Moffitt Cancer Center, the only NCI-designated comprehensive cancer center in Florida. At Moffitt, while every effort is made to recruit minorities, 85% of the women diagnosed with ovarian cancer who are treated at Moffitt are white, 7% of unknown racial and ethnic identity, 4% white Hispanic, and 3% black or African-American non-Hispanic.

This study recruited participants from the Center for Women’s Oncology in January 2010 to July 2012. Potential participants were initially identified by chart review by the Gynecologic Oncology study coordinator. Determination of potentially eligible women was made by reviewing appointment lists and medical charts; prospective participants were given a pamphlet during their visit and invited during scheduled clinic visits by the research coordinator nurse to participate. Inclusion criteria were: (1) a diagnosis of recurrent ovarian cancer (this was later modified to include women whose ovarian cancer had not yet recurred); (2) an ECOG of 2 or less with a life expectancy of at least 12 months; (3) access to transportation to travel to Moffitt; (4) willingness to participate in entire intervention; (5) ability to read, write and understand English; (6) age 18 or older; and (7) a patient being treated by a gynecologic oncologist in the Center for Women’s Oncology. The research nurse reviewed the expectations of being able to travel to Moffitt for four education sessions at which lunch would be served, and that attendance was an important part of the study; women who consented orally expressed their interest and willingness to attend the four sessions.

This study received Institutional Review Board approval and all participants completed the informed consent process. The following incentives were offered to participants: (1) $25 gift card for completed pre-test questionnaire; $25 gift card for completed post-test questionnaire; (2) lunch at each session; (3) course binder with resources tailored to each session and the following selected NCCAM booklets: “Get the Facts,” Thinking About Complementary and Alternative Medicine: A Guide for People with Cancer,” and “Paying for CAM Treatment”; and (4) for those who attended at least one session, a certificate for 1-free-service of the participant’s choice at H. Lee Moffitt’s Integrative Medicine department (value is about $60).

A purpose of the pilot was to determine feasibility of an educational intervention and obtain preliminary information to inform a larger multisite study. Based on the typical number of patients engaged in our clinic during this timeframe, we anticipated that at least 20 women (no more than 40 women) would meet our inclusion criteria and enroll as participants. We therefore did not plan to start the intervention until we had 20 consented women, and we believed that we would achieve that number within six months.

### 2.2. Intervention Design

**Figure 1:**
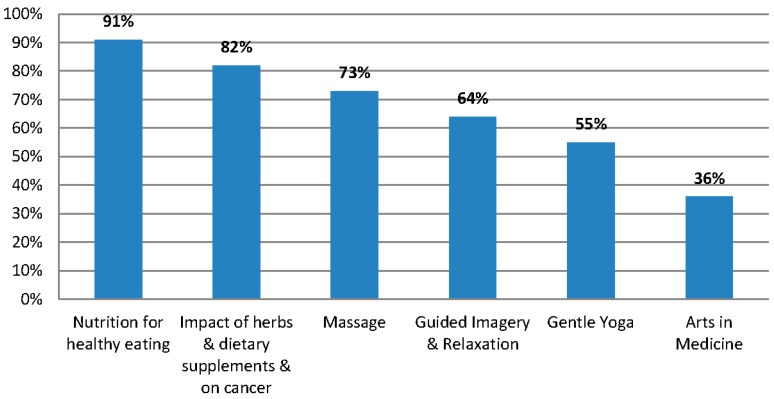
The four presentation topics were selected from six possible topics, based on pretest scores for interest level per topic. The two lower-scoring items, gentle yoga and arts in medicine, were thus not selected.

The intervention consisted of four weekly 2-h sessions that began with nutrient-dense refreshments followed by a presentation by a Moffitt CAM expert on the featured topic (Table 1). Refreshments included entrees such as chicken baked with fresh vegetables, whole grain accompaniments, fresh mixed-green salads, with optional nuts and berries, and fresh fruit. Each session was intended to be comfortably informal, combining lunch with conversation, followed by a brief introduction by a member of the study team of the guest speaker. The speaker’s 20 to 30 min presentation was followed by a question and answer session. When participants provided informed consent, they were asked to complete the baseline survey, which included a brief questionnaire that asked them to state their level of interest on several topics, as well as their preference with regard to the day and time. Topics included those that are part of Moffitt’s Integrative Medicine program; women were asked to rate their interest using a 5-point Likert scale (very interested, somewhat interested, neutral, not very interested, not at all interested) on the following: Herbs and dietary supplements and their impact on cancer treatment, nutrition recommendations for healthy eating, massage therapy, guided imagery and relaxation training, arts in medicine, and gentle yoga. Topics for each of the four sessions were the four topics with the highest interest scores, per the prospective participants, as seen in Figure 1. Specification of preferred days and times for the sessions was another contributor to attrition because unemployed women preferred mid-day, while working women preferred evening or weekends.

**Table 1:** Topics, presenters, and learning objectives for the four education sessions.

Topic	Presenter	Learning Objectives
Nutrition and Cancer: Separating Facts from Fiction	Registered Dietitian	Describe the main nutrition and lifestyle factors that can reduce cancer risk based on scientific evidence. Explain calorie density and the impact on weight management. Describe the basic components of a “Cancer Fighting Diet.”
Herbal Supplements and Their Impact on Cancer	PharmD expert on the impact of herbal supplements on cancer	List at least one supplement most commonly used by people in the US. List at least one safety concern with commonly used dietary supplements. List at least one interaction you are aware of between herbs, supplements, drugs, and disease.
The Benefits of Massage and Healing Touch	Licensed Massage Therapist	Why do we use massage for women with ovarian cancer? List at least one benefit of massage. How do you find a qualified massage therapist?
Guided Imagery and Relaxation Training	Integrative Medicine Program Leader	Be able to identify three mind-body therapies. Be able to list at least three benefits of practicing relaxation techniques and guided imagery. Be able to state at least two ways to obtain training in these techniques.

The informed consent process and baseline assessment took place in the clinic, with exceptions made for those who preferred to complete the survey at home, for whom a postage-paid pre-addressed envelope was provided. The plan for the four education sessions was to welcome the attendees and serve lunch buffet style at four small tables, with the presentation following lunch. The brief pretest was administered during lunch, and the post-test after the presentation, and questions matched each presenter’s learning objectives.

**Figure 2:**
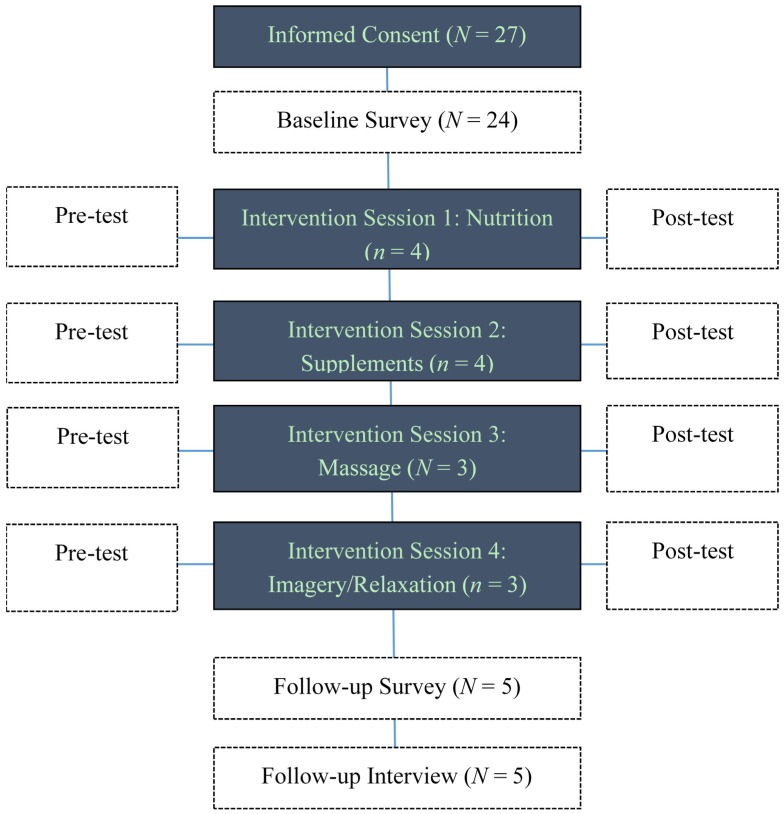
Intervention schema.

Immediately before and after each session, we administered a brief on-site pre-test. Upon completion of the intervention, participants were asked to complete a post-test. Follow-up telephone calls were made to the participants after two months from the last session. See Figure 2 for a schematic of the data collection and intervention implementation.

### 2.3. Instruments and Measures

Baseline and follow-up measures focused on QOL, prior use of CAM therapies, topic preferences, and changed intentions and behaviors associated with CAM. The baseline assessment included three validated self-report questionnaires: (1) Use of Complementary/Alternative Therapies Survey (UCATS); (2) Functional Assessment of Cancer Therapy, Ovarian (FACT-O); and (3) Memorial Symptom Assessment Scale, Short Form (MSAS-SF). Additionally, a Topic Selection Questionnaire was used once, at the beginning of the intervention, to enable women to select the four topics that are of greatest interest to them. Finally, demographic data were collected from the baseline survey, including age, race/ethnicity, employment status, residential classification, and income level. Data regarding the patient’s medical history were obtained from medical records, specifically, months from diagnosis at consent, active treatment status at consent, number of prior treatments at consent, and stage of diagnosis.

Two unvalidated, brief questionnaires were designed for this intervention: (1) Knowledge Survey; and (2) Satisfaction Survey. Three to four knowledge question based on the presenters’ learning objectives were administered before and after each intervention, educational session to assess positive changes in knowledge based on the selected topic for the day. Additionally, the Knowledge Survey and Satisfaction Survey were administered as a follow-up telephone interview eight weeks after the final session.

To assess the prevalence of use of CAM therapies, the Use of Complementary/Alternative Therapies Survey (UCATS) instrument was adapted for use in this study [25,35]. The UCATS was designed using a content validity index, resulting in an internal reliability coefficient of 0.86 for the total survey and subscales estimates of 0.67 for Diet and Nutritional Supplements, 0.79 for Stress-Reducing Techniques, and 0.80 for Traditional/Ethnic Medicines. Exploratory factor analysis was used to identify primary components (factors) embedded within the survey. A two-factor solution resulted with nine items loading heavily on a factor conceptualized as “Stress and Anxiety Reduction” and six items loading heavily on a factor conceptualized as “Dietary and Physical Manipulation.” Five items (vitamins/minerals, prayer/spiritual healing, massage, reflexology, aromatherapy) indicated moderate loadings on both factors one and two and were interpreted as equivocal items [35]. Preferences of the identified CAM therapies were ranked based on the Topic Selection Questionnaire at baseline.

Factors associated with the use of CAM therapies among women with ovarian cancer were to be identified using results from the UCATS and the Topic Selection Questionnaire, along with the Functional Assessment of Cancer Therapy-Ovarian (FACT-O) [36] and the Memorial Symptom Scale. The FACT-O is a measure of health-related QOL specific to ovarian cancer. The first part of the instrument, also referred to as the general version of the FACT, assesses four dimensions of well-being: physical, functional, social/family, and emotional. An additional subscale consisting of 13 items assesses concerns specific to ovarian cancer patients. The FACT-O has been found to have high internal consistency, good test-retest reliability, and adequate validity [36,37] The Memorial Symptom Assessment Scale-Short Form (MSAS-SF) is an abbreviated version of the MSAS. The MSAS is a validated multidimensional symptom assessment scale that measures symptom frequency, severity, and distress of 32 highly prevalent symptoms. The MSAS-SF measures each symptom in terms of distress or frequency [38].

The FACT-O and MSAS-SF were also used to assess if CAM education programs positively influenced symptom experience and QOL by comparing baseline scores to follow-up scores.

### 2.4. Data Analysis and Statistical Considerations

Because this was a pilot study, no formal inferential statistical testing was planned for this study. Statistical analyses provided preliminary information (means and standard deviations) to calculate effect sizes intended to inform power analyses for a future larger study.

The overall purpose of this preliminary study was to assess the feasibility of a larger study. Determination of feasibility included meeting all of the following criteria:

- Attendance and completion rates of ≥70% would have been seen as a positive indicator.
- A positive change in direction between pre- and post-test knowledge scores would have been interpreted as a positive indicator.
- Satisfaction scores would have been interpreted as follows: 0–2 = low, not worthy of pursuing; 2.1–3 = moderate, inconclusive and needs supportive qualitative data; 3.1–4 = high, worth pursuing on a larger scale.

## 3. Results

Recruitment spanned nearly two years, and despite vigorous efforts by more than one research nurse, only 27 women consented to participate, with just 24 women completing the baseline survey. There was turnover during that time with regard to research nurse staffing, and regretfully there is no record of how many women were approached. One woman who consented withdrew her consent because she said coming to the cancer center is stressful, and she did not want to have to come for any reason other than her necessary appointments. Reasons given for not completing the survey were provided by two consented participants: one was just told she had recurred and was too upset to focus on the survey, while another was caring for a relative who had just suffered an injury; a third did not give a reason (*n* = 3). Of the 24 women who completed the baseline survey, the mean age was 60.26 (SD 8.859); 54% were retired, 29% worked and had either a full or part time job, 12% were considered disabled, and 1 woman (4%) was unemployed (Table 2). Respondent ethnicity was 90% white, with only 1 Asian and 1 black respondent. Those from suburban communities comprised 70.8%, and 31.8% reported income between $75,000 and $100,000. The majority of the women was classified as Stage IIIC and had active treatment at the time of consent. The average number of months since diagnosis was 33 months (SD 23.33) and the average number of prior treatments was two (SD 1). On the pre- and post-tests for the few women who attended the education sessions, any blanks or incorrect answers on the pre-test were complete and correct on the post-test.

**Table 2:** Descriptive characteristics of participants who completed the baseline survey.

**Age, mean (std)**	61.0 (8.7)
**Race**	**N (%)**
White	22 (91.7%)
Asian	1 (4.2%)
Black	1 (4.2%)
**Employment status**	**N (%)**
Full-time	4 (16.7%)
Part-time	3 (12.5%)
Unemployed	1 (4.2%)
Retired	13 (54.2%)
Disabled	3 (12.5%)
**Residential Classification**	**N (%)**
Urban	3 (12.5%)
Suburban	17 (70.8%)
Small Town	4 (16.7%)
**Income Level**	**N (%)**
Under 25,000	1 (4.2%)
25,000–50,000	6 (25.0%)
50,000–75,000	6 (25.0%)
75,000–100,000	7 (29.2%)
Over 100,000	2 (8.3%)
Missing	2 (8.3%)
**Stage**	**N (%)**
IIB	1 (4.2%)
IIC	1 (4.2%)
III	1 (4.2%)
IIIC	14 (58.3%)
III/IV	1 (4.2%)
IV	6 (25.0%)
**Months from diagnosis at consent, mean (std)**	34.4 (23.9)
**Active Treatment at Consent, N (%)**	16 (66.7%)
**Number of Prior Treatments at Consent, mean (std)**	2.0 (1.2)
**Attended Intervention Sessions, N (%)**	5 (20.8%)

### 3.1. Topic and Time Preferences

Before choosing topic preferences women were asked to report their perception of their understanding of CAM. When responding to “I feel I have a good understanding of CAM”, 41% strongly agreed, 51% somewhat agreed, and 9% were not sure. The preferred topics were nutrition recommendations (91%), the impact of herbs and dietary supplements on cancer treatment (82%), massage therapy (73%), guided imagery and relaxation (64%), gentle yoga (55%), and the arts as therapy (36%) (Figure 1). Days considered to be poor choices by respondents were Monday (59%), Saturday (32%) and Thursday (27%). Most women preferred Tuesday (36%) or Wednesday (32%), with a majority wanting a presentation time of 11:30 A.M. to 2:30 P.M. (82%) *versus* 2:30 P.M. to 5:30 P.M. or after 6:00 p.m. By process of majority preferences, we opted to hold presentations on Tuesdays from 11:30 to 2:30, featuring nutrition, the impact of herbs and supplements on treatment, massage, and guided imagery.

### 3.2. Intervention Sessions

Because only those who participated in the intervention (*n* = 5) returned completed pre-/post-test surveys, time-point 2 FACT-O, MSAS, and UCATS scores are not reported. Reasons for non-participation include: died pre-intervention (*n* = 6), employment schedule conflict (*n* = 2), family care schedule conflict (*n* = 1), and declined pre-intervention (*n* = 2). The two declinations were the result of (1) injury to a family member and (2) the other had just been told she recurred and was too upset to attend. Eleven women did not provide a reason for non-participation.

The five women who participated were all white, had a mean age of 62, had an average of 15 months since diagnosis, and were all on active treatment, except for one. Three participants were stage IIIC and two were stage IV.

### 3.3. UCATS, FACT-O, and SMAS

The UCATS asked questions about individual practices associated with CAM, and most women reported that they did not use biofeedback (100%), hypnosis (96%), humor or laughter (92%), imagery (84%), acupuncture (79%), support groups (75%), yoga (63%), or meditation (58%) to obtain some kind of benefit. More than half of the women had used massage (58%), and a large majority had used prayer (87%) to obtain some kind of benefit. Only five women completed the UCATS at time 2, thus preventing a comparative analysis.

### 3.4. Follow-Up Telephone Interviews

All of the women who attended at least one education session received a telephone call within eight weeks of the final session. They were asked if they found the program helpful and whether they would attend a similar program in the future. All five replied affirmatively to both questions. When asked if during the last four weeks they had used any of the information presented in the four sessions, the following answers were provided:

- “Nutrition and meditation, which helped when resuming chemotherapy.”
- “Meditation and imagery were helpful when faced with newly diagnosed breast cancer during the program.”

When asked for comments about the program, women expressed that:

- “More attendees would have stimulated more discussion.”
- “It was held in a less-than-ideal location, an open atrium with periodic passersby.”

When asked how the program was helpful, they replied that:

- “It helped just being with other women in a similar situation.”
- “The program validated that I was doing beneficial things for myself.”
- “Although it required traveling 40 min to attend, it was worth the travel time.”

## 4. Discussion

This study developed and piloted an educational intervention regarding CAM among women with ovarian cancer. Preferred CAM topics identified and included in the educational sessions were nutrition, dietary supplements, massage therapy, and guided imagery and relaxation. Based upon the *a priori* feasibility criteria for this intervention, a larger-scaled intervention would not be advisable. However, important implications regarding intervention development and implementation for CAM among women with ovarian cancer were garnered.

### Implications for Practice

The fact that 27 women consented but only five women participated in the on-site sessions suggests that convenience is a highly important factor in planning a program. The women’s personal preferences were used to select the content of the sessions. The study initially targeted women with recurrent ovarian cancer based on the rationale that this group of women would have the greatest need for potential benefits from complementary therapies. Many of the women who consented had recurrent ovarian cancer and were too compromised by disease, too anxious about recurrent or persistent disease, or too burdened by treatment and side effects to endure travel and adherence to prescheduled sessions. As shown in Figure 2, 27 women were consented, but the intervention could not begin until there were at least 20 women enrolled. Six women had good functional status when consented, but died within weeks of being enrolled. The need to have a minimum of 20 accruals to hold the intervention sessions thus spanned two years, from 2010 through 2012. The investigators did not perceive at the outset that health would deteriorate as rapidly as it did.

Several lessons emerged from this experience. Complementary therapies have the potential to alleviate stress, increase a sense of social support, and reduce the intensity of physical discomfort [39], and introducing them solely via an on-site group intervention with rigid timing and required travel will be challenging for women whose health rapidly declines or who have schedule conflicts. Early, convenient introduction of CAM services, when health is optimal, would enable women with ovarian cancer to become informed about available services, their impact on QOL, and how to access resources and establish liaisons with CAM providers where they receive oncology care and within their community. Use of CAM therapies at first occurrence of disease may mitigate stress-related symptoms related to fear of disease recurrence [40].

An important lesson was confirmed with regard to scheduling events with fixed locations and times for women not only challenged by their disease, but also by lifestyle variables such as employment status and travel distance. While working women, who comprised a minority or those who consented, preferred evenings or Saturdays, retired or nonworking women preferred the middle of the day during the week. Future studies on CAM interventions, if on site, should be tailored for specific factors that are associated with time and distance.

While the primary intention of the CAM pilot study was education and not social support, and while pre- and post-tests for each session revealed increased knowledge, the five women who attended and completed the post-test liked the on-site group format for the feeling of support that it engendered. They liked being with women who shared their experience, but they represented just 24% of the women who had enrolled. Previous research examining sub-domains of QOL among women with ovarian cancer indicate that women with this diagnosis have higher levels of social support well-being compared to other facets of QOL (e.g., physical, functional, and emotional) [41]. The incentives for full participation ($50 for completion of two surveys, a coupon for a free service such as massage, lunch, the opportunity to learn from an expert, and a chance to be among women who were going through similar experiences) did not appear to outweigh the barriers for 76% of those who consented.

A format that provides greater accessibility is one that offers remote access, much like weekly live (but recorded for later access) teleconferences on a variety of topics presented by experts. Moffitt’s Center for Women’s Oncology, for example, hosts taped “teletalks” on a weeknight, allowing women to call in or, if they have a conflict, access the recorded teletalk at a more convenient time. The teletalk format is a 15-min presentation followed by Q&A, accessible via the Women’s Oncology website for later listening. Compared to the poor attendance at this on-site pilot, the teletalks, from 24 January 2012 to 8 October 2013, featured a multitude of topics (e.g., humor therapy, vitamins and supplements, managing fatigue, and controlling worry) with an average of 33 participants attending (range 10–81) [42]. Other potential formats for engaging women with ovarian cancer regarding CAM could be use of web-based interventions. Previous research has indicated that web-based platforms are usable and feasible among cancer survivors, especially those with limited physical mobility or who cannot attend live sessions [43]. Therefore, this type of platform may be a feasible alternative for CAM instruction, rather than a face-to-face format in this pilot.

## 5. Conclusions

CAM education should be provided early in the continuum of care, be provided in an accessible format that is tailored for individual lifestyle factors, perhaps a teleconference combined with print and website education. The location of CAM service providers, we would conjecture, may need to be more convenient, for example closer to the site where women see their physician, be able to be scheduled close to the time of the visit, and allow for consolidation of travel to accommodate more than one type of appointment at a cancer center. CAM services are known to provide benefits that can enhance quality of life for women with gynecologic and other cancers, but there is little research to guide institutions and cancer centers on formats for optimal uptake of CAM services. There is thus a need for further research on the most effective CAM education and service delivery models for women with newly diagnosed, persistent, and recurrent ovarian cancer.
